# Analysis of lung biopsies using the 2015 WHO criteria and detection of sensitizing mutations——a single-institution experience of 5032 cases

**DOI:** 10.1186/s13000-020-00975-3

**Published:** 2020-05-19

**Authors:** Yupeng Zeng, Yunxiang Zhu, Ying Ding, Liuyang Xu, Boya Zhai, Xiang Zhang, Qiaoyun Ge, Jiao Li, Qiyuan Song, Xiao Li, Zhihong Zhang

**Affiliations:** 1 Department of Pathology, the First Affiliated Hospital of Nanjing Medical University, 300 Guangzhou road, Nanjing, 210029 Jiangsu Province China; 2 Department of General Surgery, Affiliated Hospital of Yangzhou University, Yangzhou, 225001 Jiangsu Province China

**Keywords:** Lung, Biopsy, Sensitizing mutations, Epidermal growth factor receptor, Anaplastic lymphoma kinase

## Abstract

**Background:**

A specialized classification for small biopsies was added to the 2015 WHO classification of lung tumors. The purpose of this study is to explore and summarize the experience of applying the newly proposed classifications and criteria to clinical practice.

**Methods:**

We used the 2015 WHO criteria to sort out 5032 small lung biopsies from a group of Chinese patients, and demonstrated their clinicopathological features, mutational status and the relationship between these factors.

**Results:**

The most common diagnosis was primary lung carcinoma (3130, 62.2%), among which adenocarcinoma (1421, 28.2%) was the most frequent histological type. The mutational assays using ARMS-PCR technology demonstrated that *EGFR* was positive in 56.1% cases(499/889, from adenocarcinoma and NSCC, favor adenocarcinoma), *ALK* in 5.7% cases(12/211, from NSCC, which comprised all the primary lung carcinomas except small cell carcinomas), and *ROS1* in 0.9% cases(2/211, from NSCC). Another 898 NSCC specimens went through an immunohistochemical (IHC) examination for *ALK* (D5F3) and 38 of them were positive (4.2%). The overall mutation rate of *ALK* was 4.5% (50/1119). There was no significant difference between ARMS-PCR and immunohistochemistry in the positive rate of *ALK* mutation detection (*P* = 0.359). *EGFR* mutations (*P* = 0.02) and *ALK* mutations (*P* < 0.001) both decreased with an increasing patient age. Furthermore, the amount of *EGFR* mutations was higher in adenocarcinoma (64.1% vs 34.1%, *P* < 0.001) than in NSCC, favor adenocarcinoma. In contrast, *ALK* mutations were more common in NSCC, favor adenocarcinoma (4.2% vs 8.4%, *P* = 0.021).

**Conclusions:**

This single-center study exhibited a large subset of small lung biopsies from a Chinese institution and demonstrated that applying the 2015 WHO classification for small lung biopsies can help predict the mutational status of primary lung carcinomas.

## Background

According to the newly released report of cancer epidemiology in China, lung cancer is the most common malignant tumor and the leading cause of cancer death [1], which is a conclusion that can be drawn from cases worldwide [2, 3]. Unfortunately, two-thirds of the patients with lung cancer are already in the advanced stage when they are first diagnosed and thus do not qualify for surgery [4]. Their treatment options are mainly dictated by histological diagnosis based on small biopsy specimens. Furthermore, with the introduction of lung cancer screening program and advances in radiologic imaging techniques and availability, the early detection rates and the overall number of lung cancer cases identified have increased. The rapid advancement of precision lung cancer medicine has necessitated the requirement to provide complete diagnostic evaluations of small biopsy specimens. Accordingly, the 2015 WHO classification of lung tumors established the first chapter addressing the handling of small lung biopsies, and proposed corresponding criteria and terminology for the diagnosis of small biopsy specimens [5]. In this context, 5032 small lung biopsies were received by the First Affiliated Hospital of Nanjing Medical University during 2015–2018.

The purpose of this study is to explore and summarize the experience of applying the newly proposed classifications and criteria to clinical practice by clarifying the distribution of age, gender, pathologic categories and mutational status of this subset of small lung biopsies and demonstrating the relationship between these factors.

## Methods

### Patients

All the cases of small lung biopsies (bronchoscopic, needle, or core biopsies) diagnosed in the First Affiliated Hospital of Nanjing Medical University during 2015 to 2018, the total number of which was 5032, were reviewed. Simple demographic data was collected, and the diagnostics of hematoxylin and eosin, special staining and immunohistochemical (IHC) labeled sections were reviewed on all cases. Molecular pathologic testing results were reviewed on the subset of cases where these assays were performed clinically. All cases were sorted out on the basis of the diagnostic criteria and terms for small biopsies from the 2015 WHO classification of lung tumors.

### Staining

All cases were handled as routine specimens in the pathology laboratory at the First Affiliated Hospital of Nanjing Medical University. Tissues were fixed in 4% neutral buffered formalin and processed through paraffin. Two to 4 mm sections were stained with hematoxylin and eosin in addition to special stains (Acid-fast stains, PAS stains or Masson stains) and IHC labeling when clinically indicated. Table 1 lists the antibodies used for IHC. All antibodies, except *ALK* (Ventana ALK, D5F3, rabbit monoclonal anti-human *ALK*, Roche), were from Maixin Biotechnologies, Fuzhou, Fujian, China, and were used in the delivered concentrations with no need for dilution (ready-to-use). Antibody visualization was performed with the Envision Plus detection system (Ventana, Roche). Appropriate positive and negative controls were used with each antibody and each case.

**Table 1:** Details of used immunohistochemical antibodies

*Antibody*	*Clone*	*Dilution*	*Source*
TTF1	SPT24	–	Maixin, Fujian, China
Napsin-A	MX015	–	Maixin, Fujian, China
P40	ZR8	–	Maixin, Fujian, China
P63	MX013	–	Maixin, Fujian, China
CK5/6	MX040	–	Maixin, Fujian, China
CD56	MX039	–	Maixin, Fujian, China
CgA	LK2H10 + PHE5	–	Maixin, Fujian, China
Syn	SP11	–	Maixin, Fujian, China
Ki67	MX006	–	Maixin, Fujian, China
ALK	D5F3	–	Roche

### Mutational analysis

Experienced pathologists selected a block with typical morphologic features from each case to ensure sample adequacy (> 200 tumor cells). DNA was extracted from 3 to 6 sections and Amplification Refractory Mutation System PCR (ARMS-PCR) was used to establish the mutational status of *EGFR*, *ALK*, *ROS1* (AmoyDx, Xiamen, Fujian, China).

## Results

### Patient demographics

Among the 5032 small biopsies, 3280 were from men (65%) and 1752 (35%) from women. The patients age ranged from 11 to 93 years (median 63 years). The numbers of small lung biopsies each year during 2015–2018 were respectively 1068, 1299, 1511 and 1154.

### Diagnostic categories

**Fig. 1:**
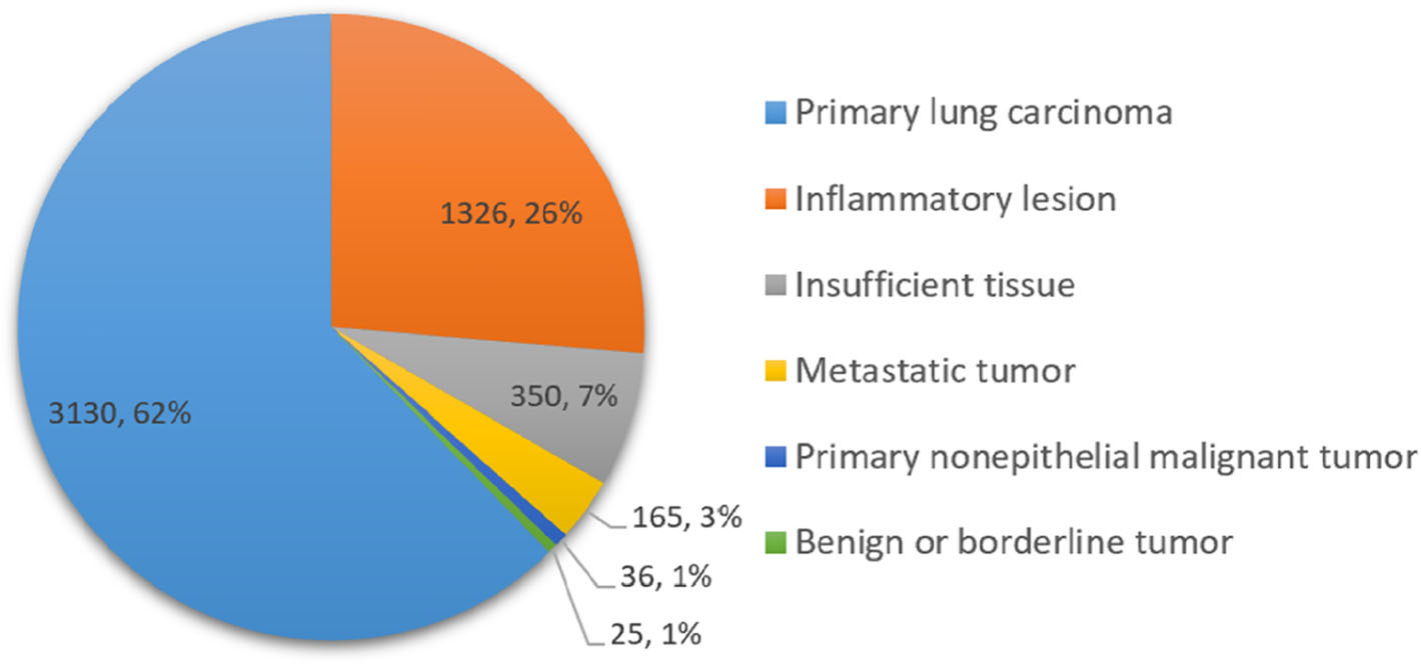
Diagnostic distribution of small biopsies

The most common diagnosis was primary lung carcinoma (3130, 62.2%), followed by inflammatory lesion (1326, 26.4%), metastatic tumor (165, 3.3%), primary nonepithelial malignant tumor (36, 0.7%), and benign or borderline tumor (25, 0.5%) (Fig. 1). Three hundred and fifty (6.9%) case had insufficient tissue or a subset of histopathologic features which were insufficient for a specific diagnosis to be rendered. A detailed classification is found in supplementary Table S1.

The majority of the 3130 primary lung carcinomas were able to be classified as squamous cell carcinoma, adenocarcinoma or small cell carcinoma (2106, 67.3%). The remaining 1024 (32.7%) lacked the characteristic morphological features of squamous cell carcinoma, adenocarcinoma or neuroendocrine tumor.

As recommended by the 2015 WHO classification schema, the following antibodies were used for IHC studies as appropriate: TTF-1, Napsin A, p40, p63, CK5/6, CD56, CgA, Syn and Ki-67 [5]. Those cases (34, 0.7%) that did not label with any antibodies or had atypical expression were classified as non-small cell carcinoma, not otherwise specified (NSCC, NOS). The remainder was further classified on the basis of their morphology and IHC labeling patterns. The diagnostic categories for all the primary lung carcinomas were summarized in Table 2.

**Table 2:** Summary of the histopathological types in 3130 cases of primary lung carcinomas

Histopathological types	Total	Rate
Adenocarcinoma	1421	45.4%
Squamous cell carcinoma	368	11.8%
Small cell carcinoma	317	10.1%
NSCC, favor adenocarcinoma	501	16.0%
NSCC, favor squamous cell carcinoma	360	11.5%
NSCC, favor typical carcinoid	6	0.2%
NSCC, favor atypical carcinoid	4	0.1%
NSCC with spindle cell and/or giant cell carcinoma	27	0.9%
NSCC, favor mixed neuroendocrine carcinoma	13	0.4%
NSCC, favor large cell neuroendocrine carcinoma	16	0.5%
NSCC, favor adenosquamous carcinoma	54	1.7%
NSCC, favor salivary gland-type tumors	9	0.3%
NSCC, NOS	34	1.1%
Total	3130	1

**Fig. 2:**
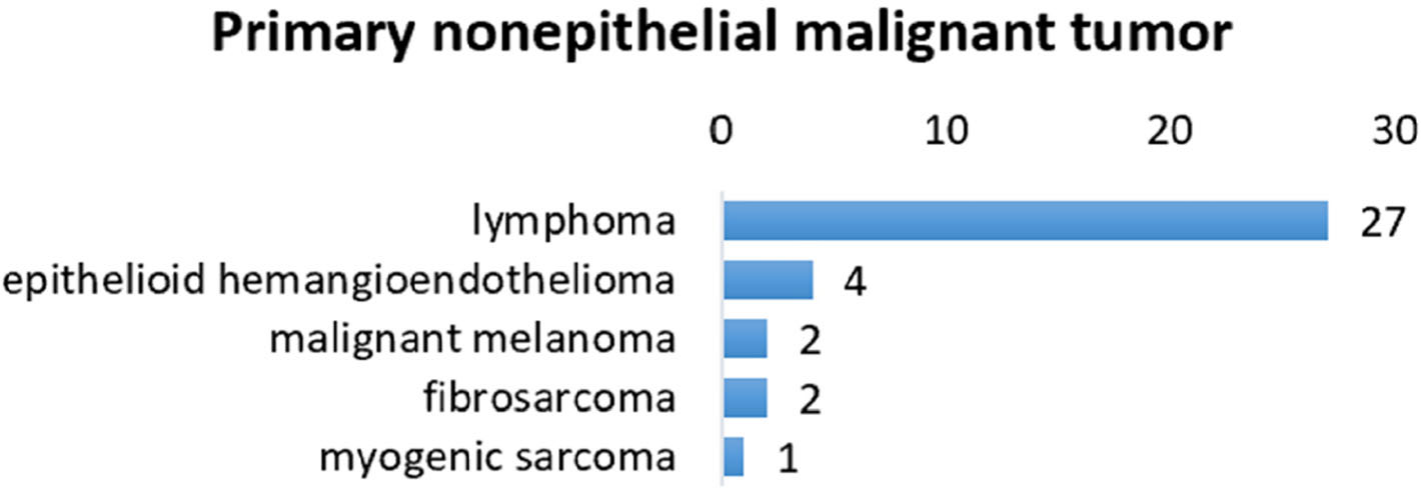
Primary nonepithelial malignant tumor

Primary non-epithelial malignant tumors occurred in 36 patients (0.7%). Twenty-seven patients were diagnosed with lymphoma, most commonly mucosa-associated lymphoid tissue lymphoma (MALToma). In addition, there were 4 cases of epithelioid hemangioendothelioma, 2 cases of fibrosarcoma, 2 cases of malignant melanoma, and 1 case of myogenic sarcoma (Fig. 2).

**Fig. 3:**
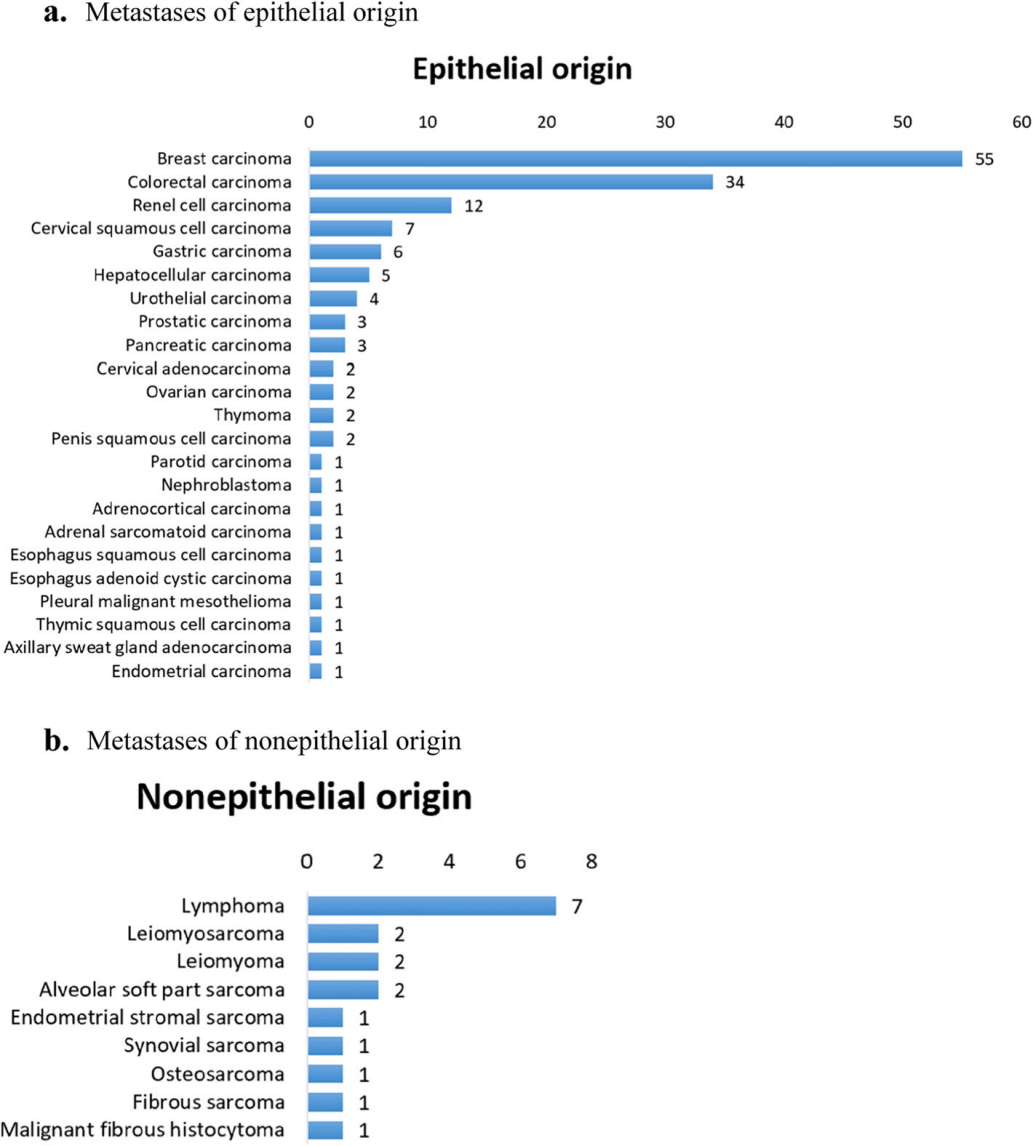
**a** Metastases of epithelial origin. **b** Metastases of nonepithelial origin

Metastatic tumors represented 165 of the cases (3.3%), 147 being of epithelial origin and 18 nonepithelial. The most common metastasis to the lung was from breast cancer (55 cases, 33.3%), followed by 34 cases (20.6%) of colorectal cancer and 12 cases (7.3%) of renal cell carcinoma (Fig. 3).

**Fig. 4:**
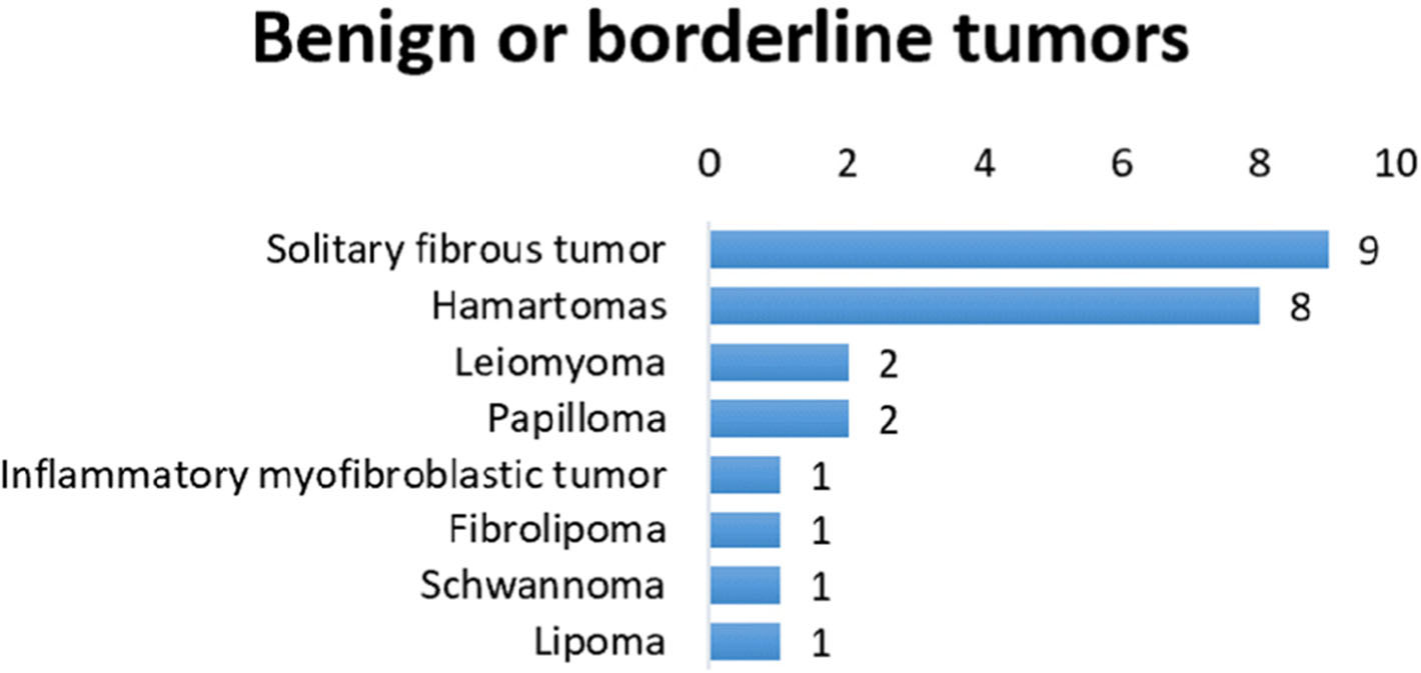
Benign or borderline tumors

Among our 5032 biopsies there were 16 benign tumors. Two cases were papillomas, considered benign of epithelial tumors. The remaining 14 benign tumors were of mesenchymal origin, including 8 hamartomas, 2 leiomyoma and 1 case each of lipoma, fibrolipoma, schwannoma, and inflammatory myofibroblastic tumor. There were also 9 solitary fibrous tumors, usually treated as a fun borderline tumor (Fig. 4).

A specific diagnosis was not achieved in 350 biopsies (6.9%), mainly due to insufficient tissue. Two samples (0.04%) were determined to be too small for processing. The microscopic appearance of 140 tissue samples demonstrated non-specific morphology such as normal lung tissue, striated muscle, dermal tissue, exudate, necrotic tissue, and red blood cell. Only descriptive diagnosis was made in these cases (2.8%). The remainder was considered as suspicious malignancy (208, 4.1%) and 3 scenarios were found where such a diagnosis should be rendered: 1) Patients with atypical cells but the atypia was inconspicuous, 2) few atypical cells were present in the tissue sample, and 3) patients with precancerous lesions, including those samples of coming from the periphery of the lesion.

### Analysis of patients undergoing surgery after biopsy

Among the 5032 cases, 444 patients underwent surgery after biopsy. A consistent diagnosis was reported for 368 cases between the surgical specimen and the biopsy specimen. The details of the remaining 76 cases, whose surgical diagnosis differed from their corresponding biopsy diagnosis, are shown in Tables 3 and 4.

**Table 3:** Comparison between the diagnoses of biopsy and surgery

Biopsy diagnoses	Surgical diagnoses	Amount	Rate
Benign lesion	Cancer	20	4.5%
Suspicious malignancy	Inflammatory lesion	3	0.7%
Suspicious malignancy	Cancer	32	7.2%
NSCC, NOS	Cancer of specific type	4	0.9%
Different in histopathological subtype		17	3.8%
Consistent in histopathological diagnostics		368	82.9%
Total		444	100.0%

**Table 4:** The 17 Cases different in subtype between surgical diagnosis and biopsy diagnosis

No.	Surgery	Biopsy
C4403	Adenosquamous carcinoma	Adenocarcinoma
C3094	Adenosquamous carcinoma	Adenocarcinoma
C2309	Adenosquamous carcinoma	Adenocarcinoma
C848	Adenosquamous carcinoma	Adenocarcinoma
C987	Adenosquamous carcinoma	Squamous cell carcinoma
C1420	Adenocarcinoma	Squamous cell carcinoma
C4319	Adenocarcinoma	Squamous cell carcinoma
C5019	Adenocarcinoma	Adenosquamous carcinoma
C14	Large cell carcinoma	Adenocarcinoma
C213	Large cell carcinoma	Squamous cell carcinoma
C1309	Squamous cell carcinoma + Sarcomatoid carcinoma	Squamous cell carcinoma
C823	Squamous cell carcinoma + Sarcomatoid carcinoma	Mixed neuroendocrine carcinoma
C2348	Squamous cell carcinoma	Spindle cell malignancy
C1048	Small cell carcinoma	Adenocarcinoma
C2947	Mucoepidermoid carcinoma	Squamous cell carcinoma
C2109	Lymphoepithelioma-like carcinoma	Squamous cell carcinoma
C1895	Lipoma	Hamartoma

### Molecular detection

Amplification Refractory Mutation System PCR (ARMS-PCR) was used to identify *EGFR* gene mutations in 889 cases of adenocarcinoma (Table 5). Four hundred and ninety-nine cases (56.1%) were found to be mutant, 238 (26.8%) with p.L858R, 222 cases (25.0%) with exon 19 deletions, 15 (1.7%) with p.L861Q, 14 (1.6%) with exon 20 insertions, 13 (1.5%) with exon 18 mutation, 13 (1.5%) with p.T790M, and 2 (0.2%) with p.S786I. There were 18 cases of co-mutation of *EGFR* (Table 6), including 8 cases of p.L858R with p.T790M, 4 cases of exon 19 deletions with p.T790M, 1 case of exon 18 mutation with p.T790M, 2 cases of p.S768I with exon 18 mutations, and 3 cases of p.L861Q with exon 18 mutations,. In this study, p.T790M and p.S768I did not occur alone.

**Table 5:** Distribution of *EGFR* mutations

Mutational type	Total	Proportion
*EGFR* L858R	238	26.8%
*EGFR* 19-del	222	25.0%
*EGFR* L861Q	15	1.7%
*EGFR* 20-ins	14	1.6%
*EGFR* Exon 18 mutant	13	1.5%
*EGFR* T790M	13	1.5%
*EGFR* S768I	2	0.2%
*EGFR* mutant	499	56.1%
*EGFR*-	390	43.9%
Total^a^	889	100.0%

^a^The number of *EGFR* mutant was less than that of *EGFR* mutations due to co-mutation

**Table 6:** The co-mutational status of *EGFR*

Mutation 1	Mutation 2	Amount
T790M	L858R	8
T790M	19-del	4
T790M	Exon 18	1
S768I	Exon 18	2
L861Q	Exon 18	3

We also assayed for *ALK* and *ROS1* mutations in 211 patients with non-small cell lung cancer through ARMS-PCR. Mutations here were identified in 12 and 2 cases respectively, corresponding to mutation rates of 5.7 and 0.9%. In addition, immunohistochemical *ALK* (D5F3) testing was performed in another 898 patients with non-small cell lung cancer. The number of positive cases and the rate of positive case were 38 and 4.2% respectively. The overall mutation rate of *ALK* was 4.5% (50/1119). There was no significant difference between ARMS-PCR and immunohistochemistry in the detection of an *ALK* mutation (*P* = 0.359).

### Analysis of the relationship between mutational status and clinicopathologic features

*EGFR* mutations were more common in females (39.7% vs 67.5%, *P* < 0.001) while no gender difference was noted in *ALK* mutations (3.7% vs 5.7%, *P* = 0.133). *EGFR* mutations (*P* = 0.02) and *ALK* (*P* < 0.001) mutations both decreased with an increasing patient age. Furthermore, the amount of *EGFR* mutations was higher in adenocarcinoma (64.1% vs 34.1%, *P* < 0.001) than in NSCC, favor adenocarcinoma. In contrast, *ALK* mutations were more common in NSCC, favor adenocarcinoma (4.2% vs 8.4%, *P* = 0.021) (Table 7).

**Table 7:** Analysis of the relationship between mutational status and clinicopathologic features

Characteristics	***EGFR***		Rate(%)	***P*** value	***ALK***		Rate(%)	***P*** value
	MT	WT			MT	WT		
Gender								
Male	221	335	39.7	**< 0.0001**	22	570	3.7	0.133
Female	283	136	67.5		23	378	5.7	
Age								
< 50	77	50	60.6	**0.020**	15	96	13.5	**< 0.0001**
50–69	306	278	52.4		27	564	4.6	
≥70	121	143	45.8		3	288	1.0	
Histological category								
Adenocarcinoma	418	234	64.1	**< 0.0001**	28	644	4.2	**0.021**
NSCC, favor adenocarcinoma	81	156	34.1		15	163	8.4	

## Discussion

Lung cancer is a malignancy with the highest incidence and mortality not only in China, but also in the United States, Europe and every other place around the world [1, 3]. For the reason that most patients of lung cancer have lost surgery opportunity at the time of diagnosis, small biopsy has become the best choice for these patients to determine the tumor histological type and molecular genetic characteristics, and to guide their follow-up treatment. CT-guided percutaneous needle biopsy was for the first time introduced to the clinicians in 1960s, and now it has gradually developed into a mature minimally invasive diagnostic technique [6]. In the meantime, with the development of endoscopic techniques, bronchoscopy has long been an important means for the diagnosis of lung cancer and one of the main methods for early detection of lung cancer [7]. In addition, in recent years, the application of diverse pathological techniques, especially immunohistochemical staining, has become increasingly mature and extensive, and the specificity and sensitivity of antibodies have been constantly improved. On the basis of this background, the postoperative diagnosis has been partially replaced by preoperative diagnosis, providing guidance for more accurate treatment. In this context, small lung biopsy is playing an increasingly important role in the diagnosis of lung cancer. This study demonstrated the distribution of a subset of 5032 small lung biopsies and revealed the clinicopathological and genetic characteristics of lung cancer from a large Chinese institution, in order to provide better assistance for clinical treatment.

We applied the criteria and terminology from the 2015 WHO classification for small lung biopsies in our study. In the previous clinical practice, a diagnosis of NSCLC was enough for the clinicians to make treatment decisions, and the diagnosis of small biopsies mostly referred to the diagnostic criteria and terminology of surgical specimens. However, the deepening of lung cancer research and the rapid development of targeted therapy have heightened the requirement for more detailed histological classification. Moreover, the diagnosis of small biopsies referring to the diagnostic criteria of surgical specimens is always not accurate and precise enough due to the heterogeneity of tumor tissues and the randomness of biopsy technique. As a result, the 2015 WHO classification of lung tumors for the first time provided a specialized classification for small biopsies (bronchoscopic, needle, or core biopsies).

The latest WHO classification emphasized the importance of saving specimens in the diagnostic process of small lung biopsies. In addition to making H&E sections and immunohistochemical sections, we should set aside adequate specimens for molecular pathologic detection to guide targeted therapy. For patients with clearly defined adenocarcinoma differentiation, patients with poor morphological differentiation and immunophenotypic tendency toward adenocarcinoma, patients with large cell carcinoma as well as patients with NSCC, NOS, the testing of *EGFR*, *ALK*, *ROS1* and other tumor driver genes should be performed to help find probable therapeutic targets. The specialized terms for small biopsies are roughly in correspondence with those of surgical specimens, while the former feels more conciliatory. Those cases which are obviously inconsistent with small cell carcinoma in morphology and do not have the typical morphological features of adenocarcinoma or squamous cell carcinoma should be firstly classified as non-small cell carcinoma (NSCC). Then, a diagnostic tendency should be defined on basis of morphological features and immunophenotypic characteristics, such as “favor adenocarcinoma”, “favor squamous cell carcinoma”, “with spindle cell and/or giant cell carcinoma”, and so forth. Some cases lack typical morphological features of squamous cell carcinoma, adenocarcinoma or neuroendocrine tumor while their immunophenotype is atypical or even completely naked. This part of cases should be divided into non-small cell carcinoma, not otherwise specified (NSCC, NOS). If these cases still do not have typical morphology and specific immunostaining after radical resection and adequate sampling, they will be diagnosed as large-cell carcinoma. Pathologists should pay attention that, due to the limited amount of tissue, it could be very hard to determine the origin of a malignancy merely with small biopsy specimens. Therefore, the substitution of non-small cell carcinoma (NSCC) for non-small cell lung cancer (NSCLC) can make the diagnosis more accurate and cautious. The diagnostic terms of small lung biopsies provided by the latest version of the WHO classification are more moderate and cautious, and simultaneously correspond one-to-one to those from the diagnostic classification of surgical specimens. In this way, pathologists can render diagnoses based on both small biopsies and surgical specimens more flexibly. In the meantime, clinicians and patients can have a better understanding of the randomness and limitations of small biopsies.

The lung is the only organ that receives blood and lymph circulation from the whole body. The dense capillary network in the lung is the first barrier for tumor cells to enter the venous system from the lymphatics [8]. Therefore, metastatic tumors are more common in lung than in other organs, and tumors originating from almost any part of the body can metastasize to the lung. In this study, there were 165 cases of metastatic tumors, the most common metastasis to lung was from breast carcinoma (55 cases, 33.3%), followed by 34 cases (20.6%) of colorectal carcinoma and 12 cases (7.3%) of renal cell carcinoma. According to the data from International Registry Lung Metastases and another similar study of Indian population, the top three lung metastatic tumors were colorectal carcinoma (33–37%), breast carcinoma (17–19%), and renal cell carcinoma (12–17%) [9–12]. Actually, the global incidence of colorectal cancer is lower than that of breast cancer [3]. Therefore, it can be speculated that colorectal cancer is more likely to metastasize to the lung than breast cancer, and breast cancer is more likely to metastasize to the lung in the Chinese population than in others.

It is difficult to determine the origin of tumor only by small lung biopsies. Usually, pathologists can provide clinicians with some clues based on H&E morphology and immunostaining. Distinguishing pulmonary enteric adenocarcinoma (PEAC) from metastatic colorectal adenocarcinoma (MCAC) is of particular concern. Both of PEAC and MCAC can be present with a morphology of colorectal carcinoma, and CK20, CDX2 and MUC2 can be immunohistochemically positive in both of them. Usually, MCAC does not have other morphological subtypes except enteric morphology, and CK7, TTF-1 and Napsin A are generally negative in MCAC [13]. In rare cases, the MCAC can express TTF-1, where it may not be completely distinguished from PEAC [14]. Therefore, for the patients with a history of colorectal cancer and a lung lesion of microscopical enteric morphology, a diagnosis of MCAC is a priority. Currently, only after MCAC is clinically excluded by colonoscopy can a pathologist render a diagnosis of primary PEAC. Recent studies have shown that PEAC demonstrates similar mutational characteristics to non-small cell lung cancer, rather than to primary or metastatic colorectal adenocarcinoma [14].

The proportion of adenocarcinoma, squamous cell carcinoma and small cell carcinoma was slightly higher than that of Asian population from SEER database, which were 61.4% vs 58.1, 23.3% vs 15.5, 10.1% vs 7.8% respectively. We can find that adenocarcinoma account for the majority of primary lung cancer in our study, which has been a growing trend in recent decades.

In this study, 444 patients underwent surgery after small biopsy, among which 368 patients received a consistent diagnosis. These cases made up a concordance rate of 82.9%, without treatment option changes. Among the 17 cases that received different histopathological diagnostics based on the surgery after small biopsy, the most common situation was that adenosquamous carcinoma was misdiagnosed as adenocarcinoma (4 cases) or squamous cell carcinoma (1 case) in the previous diagnosis. These cases fully demonstrated the heterogeneity of lung cancer and the randomness and limitations of small biopsy. Therefore, as pathologists, we should be aware that we can never be too scrupulous when dealing with small lung biopsies.

The frequency of *EGFR* mutation in this study was 56.1%, which was close to that of the Chinese population, 50.2%, reported by PIONEER study [15]. According to previous studies and this one, we can find that the frequency of *EGFR* mutation in China and other countries/regions in East Asia (approximately 30–64%) [15–17] is significantly higher than that in India (22.2%) and among white people (approximately 20%) [18]. We found that the amount of *EGFR* mutations was significantly higher in female adenocarcinoma patients than in male, which was also supported by the data from PIONEER study. It was demonstrated in our study that frequency of *EGFR* mutation in well-differentiated adenocarcinoma was significantly higher than that in poorly-differentiated adenocarcinoma, while PIONEER study showed that the mutational rate of *EGFR* was significantly higher in invasive adenocarcinoma than in bronchioloalveolar carcinoma, which is currently called pulmonary adenocarcinoma in situ. Therefore, it can be speculated that the amount of *EGFR* mutation is higher in invasive adenocarcinoma with distinct morphological differentiation, which needs to be confirmed through further research.

The previously reported prevalence of *ALK* mutation was 3–7% [19–22]. In this study, *ALK* mutation was found positive in 5.7% cases through ARMS-PCR and in 4.2% cases through IHC(D5F3), which was slightly lower than the mutational rate of 6.1% detected by Wang through IHC(D5F3) [23]. The overall frequency of *ALK* mutation was 4.5% in this study, which was almost consistent with previous reports. The frequency of *ROS1* mutation was 0.9% in this study, which was slightly lower than that in previous reports (1.2–2.2%) [24–29], probably because of the small amount of our ROS1-tested cohort.

## Conclusions

This single-institution study demonstrated the distribution of a large number of small lung biopsies and revealed the clinicopathological and genetic characteristics of lung cancer from a large Chinese institution. On the basis of that, we found *EGFR* mutations were more common in females, younger age groups, and well-differentiated adenocarcinoma. Simultaneously, *ALK* mutation tended to be more common in older age groups, poorly-differentiated adenocarcinoma, and had no gender difference. The criteria and terminology provided by the 2015 WHO classification for small lung biopsies can help predict the mutational status of primary lung carcinomas, and they should be applied to pathologists’ daily work.

## Supplementary information

**Additional file 1: Supplementary Table 1.** The histopathological distribution of the 5032 cases of small lung biopsies.

## Data Availability

Data and materials of this work are available from the corresponding author on reasonable request.

## Abbreviations

ALK: Anaplastic lymphoma kinase
ARMS-PCR: Amplification refractory mutation system-polymerase chain reaction
EGFR: Epidermal growth factor receptor
IHC: Immunohistochemistry
MCAC: Metastatic colorectal adenocarcinoma
NSCC: Non-small cell carcinoma
NSCC, NOS: Non-small cell carcinoma, not otherwise specified
NSCLC: Non-small cell lung cancer
PEAC: Pulmonary enteric adenocarcinoma
WHO: World Health Organization
