# Supplementary material for: Analysis of lung biopsies using the 2015 WHO criteria and detection of sensitizing mutations——a single-institution experience of 5032 cases
Source: Diagn Pathol. 2020 May 19;15:59. doi: 10.1186/s13000-020-00975-3 (PMC7236924; doi:10.1186/s13000-020-00975-3)
Supplement: Supplementary file 1 — Additional file 1: Supplementary Table 1. The histopathological distribution of the 5032 cases of small lung biopsies. [file 13000_2020_975_MOESM1_ESM.docx]

**Supplementary table 1.** The histopathological distribution of the 5032 cases of small lung biopsies

| Histopathological types | 2015 | 2016 | 2017 | 2018 | Total | Ratio |
| --- | --- | --- | --- | --- | --- | --- |
| Adenocarcinoma | 255 | 376 | 439 | 351 | 1421 | 28.2% |
| NSCC, favor adenocarcinoma | 150 | 111 | 138 | 102 | 501 | 10.0% |
| Squamous cell carcinoma | 73 | 90 | 123 | 82 | 368 | 7.3% |
| NSCC, favor squamous cell carcinoma | 66 | 107 | 95 | 92 | 360 | 7.2% |
| Small cell carcinoma | 81 | 81 | 87 | 68 | 317 | 6.3% |
| NSCC, favor typical carcinoid | 1 | 1 | 2 | 2 | 6 | 0.1% |
| NSCC, favor atypical carcinoid | 0 | 0 | 1 | 3 | 4 | 0.1% |
| NSCC with spindle cell and/or giant cell carcinoma | 3 | 10 | 8 | 6 | 27 | 0.5% |
| NSCC, favor mixed neuroendocrine carcinoma | 2 | 3 | 4 | 4 | 13 | 0.3% |
| NSCC, favor large cell neuroendocrine carcinoma | 0 | 5 | 8 | 3 | 16 | 0.3% |
| NSCC, favor adenosquamous carcinoma | 11 | 8 | 19 | 16 | 54 | 1.1% |
| NSCC, favor salivary gland-type tumors | 0 | 4 | 4 | 1 | 9 | 0.2% |
| NSCC, NOS | 10 | 14 | 6 | 4 | 34 | 0.7% |
| Malignant mesenchymal tumor | 3 | 3 | 2 | 1 | 9 | 0.2% |
| Lymphoma | 5 | 8 | 10 | 4 | 27 | 0.5% |
| Metastatic tumor | 36 | 49 | 43 | 37 | 165 | 3.3% |
| Benign or borderline tumors | 4 | 7 | 8 | 6 | 25 | 0.5% |
| Inflammatory lesion | 291 | 343 | 397 | 295 | 1326 | 26.4% |
| Suspicous malignancy | 47 | 47 | 67 | 47 | 208 | 4.1% |
| Descriptive diagnosis | 30 | 31 | 49 | 30 | 140 | 2.8% |
| Too small tissue for processing | 0 | 1 | 1 | 0 | 2 | 0.0% |
| Total | 1068 | 1299 | 1511 | 1154 | 5032 | 1 |
